# A Review of the Oral Semaglutide in Adults with Overweight or Obesity (OASIS) Trials Evaluating Oral Semaglutide (Wegovy) for Chronic Weight Management in Adults With Overweight or Obesity

**DOI:** 10.7759/cureus.107970

**Published:** 2026-04-29

**Authors:** Blessing T Ojinna, Sara Tariq, Osamede Agho, Rabia Channa, Gurdeep Singh

**Affiliations:** 1 Internal Medicine, Guthrie Lourdes Hospital, Binghamton, USA; 2 Global Health, Emory University Rollins School of Public Health, Atlanta, USA; 3 Internal Medicine, Igbinedion University Medical School, Benin, NGA; 4 Endocrinology, Diabetes, and Metabolism, Guthrie Lourdes Hospital, Binghamton, USA

**Keywords:** obesity, oral semaglutide, overweight, wegovy, weight loss

## Abstract

Obesity is an increasingly growing public health issue affecting a substantial proportion of the population in the United States. This condition increases the risk for multiple disease processes, including cardiometabolic disorders, osteoarthritis, gout, respiratory issues, reproductive problems, and some cancers. Glucagon-like peptide-1 (GLP-1) receptor agonists got their first US Food and Drug Administration (FDA) approval in 2005 in the form of exenatide for type 2 diabetes mellitus (T2DM) management. Thereafter, GLP-1 analogs have been increasingly studied as a noninvasive strategy for weight reduction, and they transitioned from diabetes-only therapies to weight management with the approval of once-daily liraglutide prior to weekly semaglutide formulation in 2021. In essence, GLP-1 receptor agonists have emerged as a promising drug for long-lasting weight reduction and blood glucose control for patients with or without type 2 diabetes mellitus, surpassing traditional methods such as lifestyle changes and bariatric surgery.

Oral Wegovy (Rybelsus) was approved for T2DM management in 2020; however, it is approved for weight loss after the recent publication of Oral Semaglutide in Adults with Overweight or Obesity (OASIS). This literature review will discuss and summarize weight loss results from OASIS trials 1, 2, 3, and 4. The OASIS 1 and 2 trials compared the therapeutic efficacy and clinical safety profile of once-daily oral semaglutide 50 mg versus placebo on overweight and obese participants without T2DM and both with and without T2DM, respectively. The OASIS 3 trial assessed the therapeutic efficacy and clinical safety profile of once-daily oral semaglutide 50 mg versus placebo in 200 Chinese adults who were obese and overweight with one or more weight-related comorbidities, including T2DM. The OASIS 4 trial evaluated the safety and effectiveness of once-daily semaglutide 25 mg in obese and overweight patients without T2DM. Clinical trial data and relevant literature were retrieved through database searches of the National Center for Biotechnology Information and ClinicalTrials.gov. The OASIS 1, 2, and 4 demonstrated that oral semaglutide has superior efficacy compared to placebo in body weight loss, which helped secure FDA approval for oral Wegovy. Though OASIS 3 is registered in clinical trials, the results of OASIS 3 have not been published in a peer-reviewed journal yet.

## Introduction and background

Overweight and obesity have become one of the 21st century's most significant and rapidly growing public health issues. An estimated 2.5 billion people worldwide are classified as overweight, with approximately 890 million individuals meeting the criteria for obesity, a condition whose prevalence has more than doubled since 1990. Approximately 43% of individuals globally were overweight, and 16% were obese in 2022, highlighting the significance of excess body weight as a global risk factor for chronic diseases [1].

The epidemic is equally severe in the United States. According to recent national health data, more than 40% of adults suffer from obesity, and about 10% have severe obesity. This condition increases the risk for multiple disease processes, including cardiometabolic disorders, osteoarthritis, gout, respiratory issues, reproductive problems, and some cancers [2,3].

In addition to negatively impacting an individual's health, the high prevalence of excess body fat accumulation incurs substantial financial costs due to increased healthcare utilization, lost productivity, and long-term disability. In 2019, overweight and obesity were estimated to cost 2.19% of global gross domestic product (GDP), with the economic burden varying widely from about US$20 per person in Africa to US$872 per person in the Americas [4]. In 2016, it was estimated that in the United States, the total adult medical spending attributable to obesity reached US$260.6 billion nationwide [5].

Although guidelines for treating overweight and obesity in the United States recommend traditional methods of managing weight, such as lifestyle changes and behavioral therapy as the first steps in management, these approaches only yield modest population-level outcomes, and the maintenance of any accomplished weight loss proved difficult [6].

Treatment with medications is recommended for individuals with a body mass index (BMI) of >30 or a BMI of >27 in the presence of comorbid conditions [7]. Several medications, for example, phentermine-topiramate, naltrexone-bupropion, orlistat, dual-action glucagon-like peptide-1 (GLP-1)/glucose-dependent insulinotropic polypeptide (GIP) agonists, and GLP-1 receptor agonists, have been approved for this. However, GLP-1 receptor agonists such as semaglutide produce more clinically significant, long-lasting weight loss when compared to other agents [8].

Semaglutide is distributed by Novo Nordisk using the trade names Rybelsus, Ozempic, and Wegovy for the management of type 2 diabetes (T2D) and obesity. The primary distinctions among these branded products involve differences in dosage, the route of administration, and approved indications. Rybelsus is taken orally once a day for type 2 diabetes mellitus (T2DM) management in adults. It is available in 3 mg, 7 mg, and 14 mg tablets. Ozempic is an injectable form of semaglutide for the management of type 2 diabetes. It is supplied in prefilled, self-injectable pens and administered once weekly, with available doses ranging from 0.25 mg to 2 mg. Injectable Wegovy (semaglutide), on the other hand, is used in treating weight issues for individuals aged 18 and above with overweight or obesity with a minimum of one weight-associated comorbidity, such as type 2 diabetes, dyslipidemia, or hypertension. Wegovy is injected subcutaneously once per week at a higher dose of 2.4 mg using a prefilled pen [9].

Until recently, the approved GLP-1 agonists were provided by subcutaneous (SC) injection, which has created ongoing obstacles to patient acceptance, cost, and convenience. The initial dose titration and follow-up phase for injectable GLP-1 therapies may impede adherence, limit access in resource-constrained settings, and increase overall treatment costs [7].

In terms of managing chronic weight, the development of oral Wegovy (semaglutide) may represent a fundamental change in how the medication is administered. Oral Wegovy offers efficacy comparable to injectable formulations and is supported by the Oral Semaglutide in Adults with Overweight or Obesity (OASIS) 1 and 2 programs, including OASIS 4, which shows a mean weight loss of over 15% with daily oral semaglutide compared to placebo over 68 weeks in people with overweight or obesity and comorbidities [10].

By offering a tablet alternative, this treatment could overcome several practical challenges related to injections. It will make dosing without needles easier, may reduce barriers to patient uptake and prescribing, and has the potential to improve compliance and expand access through competitive pricing. The oral formulation's early US launch pricing is lower than that of many injectable regimens, indicating a greater likelihood that healthcare systems and cost-conscious individuals can implement successful long-term obesity management techniques [7].

This review will examine the OASIS clinical data for oral Wegovy, place it in the context of the obesity epidemics in the United States and around the world, and investigate how this innovative oral strategy can help overcome the drawbacks of injectable medications to achieve fair, sustainable weight management.

## Review

Methodology

We conducted a database search of the National Center for Biotechnology Information and Google Scholar using the MeSH terms "oral semaglutide," "overweight," and "obesity" for studies published from January 2000 to January 2026. This search yielded 224 results with full text available in English. We further refined the search by including "OASIS (Oral Semaglutide in Adults with Overweight or Obesity)," which resulted in five articles. Of these, we selected three original randomized controlled clinical trials for review. Additionally, we included a yet-to-be-published trial from OASIS 3 identified through ClinicalTrials.gov. Our review focuses on these four studies to provide insight into the benefits of oral semaglutide for chronic weight loss. We excluded articles on injectable semaglutide and randomized/non-randomized controlled trials of oral semaglutide that did not focus on weight management.

Summary of trials (OASIS 1, OASIS 2, OASIS 3, and OASIS 4)

*Summary of OASIS 1 Tri* *al Evidence*

OASIS 1 is a phase 3 trial evaluating superiority in a randomized, double-blind, placebo-controlled design conducted at 50 ambulatory healthcare centers in nine countries spanning Asia, Europe, and North America. The study included eligible male and female participants ages 18 and above (20 years and above in Japan, in accordance with regulatory requirements) having a BMI of 30 kg/m², or at least 27 kg/m², along with at least a health condition related to excess weight and, at a minimum, one attempt to lose weight through diet. Of the participants, 485 (73%) were women, and 182 (27%) were men. Major exclusion criteria were self-reported weight changes exceeding 5 kg within a 90-day period prescreening, prior or intended bariatric surgery, a glycated hemoglobin (HbA1c) of 6.5% or higher at screening, and a history of diabetes. The recent OASIS 1 clinical trial evaluated the effectiveness and safety profile of the oral semaglutide 50 mg, administered once daily, relative to placebo for the management of obesity or overweight among adults without type 2 diabetes [10].

Subjects enrolled in the study were distributed 1:1 via an interactive web-based randomization system to receive either oral semaglutide, titrated to 50 mg daily, or an identical-appearing placebo, both for 68 weeks, alongside lifestyle intervention. The study was double-blinded, with participants, investigators, and outcome assessors unaware of group assignments. The coprimary endpoints were the percent difference in body weight and the proportion of participants achieving no less than 5% weight loss at week 68 with oral semaglutide 50 mg compared to placebo. These outcomes were assessed using an intention-to-treat analysis, regardless of treatment discontinuation or the use of other weight loss therapies. Overall, 667 participants were assigned at random to receive either oral semaglutide 50 mg (n=334) or placebo (n=333). Oral semaglutide treatment began at 3 mg, with the dose increased every four weeks or longer if necessary for tolerance to 7 mg, 14 mg, and 25 mg and eventually sustaining a dose of 50 mg by week 16. If a participant was unable to tolerate the 50 mg maintenance dose, a lower dose could be prescribed as determined by the investigator, with a minimum of one recommended attempt to titrate back to 50 mg [10].

Results showed that mean body weight declined in both treatment groups over the course of the trial. For the "treatment policy estimand" (actual treatments received), which assesses the impact of the intervention guided by the actual treatments received by the participants, at week 68, the estimated mean change from baseline was -15.1% (standard error {SE}: 0.5) for the participants receiving oral semaglutide 50 mg, compared to -2.4% (SE: 0.5) for those receiving placebo (coprimary outcome measures). For the "trial product estimand" (taken exactly as intended), which evaluates the effect of the treatments if taken exactly as intended, the estimated average difference in body weight at week 68 was -17.4% (SE: 0.5) with oral semaglutide 50 mg, compared to -1.8% (SE: 0.5) with placebo. During the trial observation window, 269 of 317 participants (85%) in the oral semaglutide 50 mg category experienced at least a 5% reduction in body weight by week 68, compared to 76 of 295 participants (26%) in the placebo group. For the on-treatment observation period, these numbers were 247 of 277 (89%) for oral semaglutide and 60 of 245 (24%) for placebo.

The likelihood of attaining at least 5% weight loss was significantly higher with oral semaglutide 50 mg than with placebo for both the treatment policy (coprimary outcome measures; odds ratio {OR}, 12.6; 95% confidence interval {CI}, 8.5-18.7; p<0.0001) and trial product (OR, 55.2; 95% CI, 33.0-92.4) estimands. The participants treated with oral semaglutide 50 mg were also significantly more likely than those receiving placebo to achieve at least 10%, 15%, and 20% reductions in body weight. These greater weight reductions coexisted with notable benefits using participant-reported data, including Impact of Weight on Quality of Life-Lite Clinical Trials Version (IWQOL-Lite-CT) physical function and Short Form (SF)-36v2 physical functioning scores, for both estimands. These confirmatory secondary endpoints comprised the fraction of participants achieving 10%, 15%, or 20% or more weight loss, as well as changes in physical function scores. Additional supportive secondary endpoints for both the treatment policy and trial product estimands demonstrated favorable outcomes with 50 mg oral semaglutide versus placebo. This encompassed reductions in cardiometabolic factors such as BMI, total body weight (kg), abdominal circumference, and improvements in blood pressure readings, glycemic control (HbA1c, fasting glucose level, and fasting serum insulin), fasting lipid profiles, and ultrasensitive C-reactive protein.

OASIS 1 trial demonstrated that among adults with obesity or overweight, tablet semaglutide 50 mg once daily, when combined with diet and physical activity, was significantly more effective than placebo in producing substantial body weight reduction and enhancing physical functioning. Notably, over two-thirds of the participants in the semaglutide group achieved a minimum of 10% weight loss, greater than half lost no less than 15%, and approximately one-third experienced reductions of a minimum of 20%, all rates that surpassed those observed with placebo [10].

Safety:* *The safety profile of oral semaglutide 50 mg closely resembled that of 2.4 mg subcutaneous semaglutide, as well as the broader GLP-1 receptor agonist class [10]. The rate of the premature discontinuation of the trial product was seen in 47 participants, the same as that identified with 2.4 mg subcutaneous semaglutide of the STEP 1 trial [11]. The predominantly reported adverse events with tablet semaglutide 50 mg were gastrointestinal (GI) in nature, such as nausea, constipation, diarrhea, and vomiting, as well as COVID-19. Digestive system side effects generally resolved quickly, with mild to moderate intensity, and typically resolved without requiring the persistent discontinuation of the study drug. The incidence of digestive system symptoms was greatest during the dose up-titration phase. Table 1 summarizes safety outcomes in this trial.

**Table 1:** Adverse events in the OASIS 1 trial Table adapted from OASIS 1 by Blessing Ojinna [10] *Event rate reported as events per 100 patient-years (as reported in trial tables) †Data are from the in-trial observation period, defined as the length of time between randomization and last site contact, irrespective of whether the participants discontinued the trial product or received other body weight-lowering therapies (such as medications or bariatric surgery) n, number of participants; OASIS, Oral Semaglutide in Adults with Overweight or Obesity

Adverse event	Semaglutide 50 mg tablet (n=334)	Semaglutide 50 mg tablet (n=334)	Semaglutide 50 mg tablet (n=334)	Placebo (n=333)	Placebo (n=333)	Placebo (n=333)
	Participants with ≥1 event, n (%)	Event number	Event rate*	Participants with ≥1 event, n (%)	Number of events	Event rate*
Any adverse event	307 (92%)	2500	561.3	285 (86%)	1577	366.7
Serious adverse events	32 (10%)	44	9.9	29 (9%)	48	11.2
Adverse events leading to trial product discontinuation	19 (6%)	27	6.1	12 (4%)	17	4.0
Gastrointestinal disorders, leading to discontinuation	12 (4%)	19	4.3	5 (2%)	7	1.6
Fatal events†	0 (0%)	-	-	0 (0%)	-	-
Hypoglycemia†	3 (1%)	4	0.8	1 (0%)	1	0.2
Any gastrointestinal disorder	268 (80%)	1136	255.1	154 (46%)	382	88.8
Some adverse events reported in >5% of the participants	Some adverse events reported in >5% of the participants	Some adverse events reported in >5% of the participants	Some adverse events reported in >5% of the participants	Some adverse events reported in >5% of the participants	Some adverse events reported in >5% of the participants	Some adverse events reported in >5% of the participants
Nausea	173 (52%)	331	74.3	51 (15%)	64	14.9
COVID-19	120 (36%)	128	28.7	116 (35%)	122	28.4
Constipation	92 (28%)	123	27.6	50 (15%)	71	16.5
Diarrhea	89 (27%)	169	37.9	56 (17%)	70	16.3
Vomiting	80 (24%)	154	34.6	12 (4%)	14	3.3
Decreased appetite	56 (17%)	61	13.7	24 (7%)	26	6.0
Dyspepsia	47 (14%)	64	14.4	17 (5%)	19	4.4
Headache	46 (14%)	80	18.0	29 (9%)	36	8.4
Nasopharyngitis	38 (11%)	54	12.1	49 (15%)	73	17.0
Upper respiratory tract infection	19 (6%)	26	5.8	20 (6%)	29	6.7
Urinary tract infection	19 (6%)	27	6.1	8 (2%)	8	1.9
Back pain	16 (5%)	20	4.5	24 (7%)	37	8.6
Hypertension	10 (3%)	12	2.7	22 (7%)	24	5.6

Summary of OASIS 2 Trial Evidence

The OASIS 2 trial was a 68-week, multicenter, double-blind, placebo-controlled phase 3a randomized clinical study with an additional seven-week follow-up period. It was conducted between November 2021 and September 2023 at nine sites in Japan and four sites in South Korea. Enrolled individuals comprised adults aged 20 years or older in Japan with a BMI of 27.0 or higher plus two or greater obesity-connected sequelae or a BMI of 35.0 or higher with at least one complication. Each participant was required to have at least one complication, such as hypertension, dyslipidemia, or type 2 diabetes (T2D). Around 25% of participants were expected to have type 2 diabetes at screening. Of the 201 participants enrolled, 87 (43.3%) were women, and 114 (56.7%) were men.

For participants with diabetes, additional eligibility criteria included a glycated hemoglobin (HbA1c) level within the range of 7.0%-10.0% at screening, a diagnosis of T2DM made a minimum of 180 days prior to screening, and dietary or exercise control only or stable therapy with up to three oral glucose-lowering medications. All participants also had a history of no fewer than one failed self-reported effort at weight loss. Major exclusion criteria were the recent use of weight management medications in the 90 days leading up to screening, a history of or scheduled obesity surgery over the course of the study period, and reporting a weight fluctuation of 5 kg or more in the 90 days leading up to screening. The OASIS 2 clinical trial evaluated the effectiveness and tolerability profile of once-a-day semaglutide 50 mg orally for treating obesity and overweight in East Asian adults, in the presence or absence of type 2 diabetes [12]. The Japanese Society for the Study of Obesity (JASSO) and the Korean Society for the Study of Obesity (KSSO) define obesity as a BMI of 25 or higher. Both organizations recommend considering pharmacotherapy for individuals who are unable to meet weight-reduction goals through nutrition, exercise, and behavioral therapy alone [13,14].

Eligible participants were allocated in a 2:1 ratio to receive once-daily oral semaglutide 50 mg or placebo using an interactive web platform. (Calyx and Perceptive) with stratification by T2D diagnosis and planned computed tomography (CT) scan. The appearance and packaging of oral semaglutide and placebo were indistinguishable. Both participants and study investigators were masked to treatment assignment throughout the study. All study participants underwent a health-promoting intervention that incorporated counseling from a nutritionist or another licensed healthcare provider. Dietary advice targeted a daily calorie deficit of 500 kcal, along with a recommendation to perform no less than 150 minutes of exercise each week. The participants were administered semaglutide orally or a placebo once daily, starting at 3 mg and escalating every four weeks to 7 mg, 15 mg, 25 mg, and ultimately 50 mg by week 16. The long-term dose was continued for 52 weeks until week 68, followed by a seven-week follow-up period. A subset of Japanese participants, including up to 25% with type 2 diabetes, received CT scans both at baseline and at week 68 to measure changes in body fat distribution, specifically visceral adipose tissue area and subcutaneous adipose tissue area [12].

Results:* *Overall, 182 out of 201 participants (90.5%) completed the treatment phase: 118 of 134 (88.1%) for those assigned to oral semaglutide and 64 of 67 (95.5%) in the cohort receiving placebo. A total of 194 participants (96.5%) completed the entire trial. Of those who finished oral semaglutide treatment, 96 of 118 (81.4%) were taking the 50 mg dose at week 68. The coprincipal outcomes were defined as the body weight percent difference and the fraction of study subjects attaining a minimum of 5% decrease from baseline. Confirmatory secondary endpoints (included in the statistical testing hierarchy) comprised the fraction of participants dropping at least 10% of their pre-treatment body weight and the variation in the functional ability score of the Impact of Weight on Quality of Life-Lite Clinical Trials Version (IWQOL-Lite-CT) supportive secondary endpoints included differences in body weight, BMI, abdominal circumference, systemic blood pressure, HbA1c, fasting lipid levels, and high-sensitivity C-reactive protein (hsCRP). Additional supportive secondary endpoints were the fraction of participants with a minimum of 15% or 20% weight loss, variation in glycemic status, and the percentage and absolute change in visceral adipose tissue area and subcutaneous adipose tissue area among Japanese participants who underwent CT scans. Between baseline and week 68, the participants experienced a mean standard error of mean (SEM) reduction in weight of -14.3% (0.8) with oral semaglutide compared to -1.3% (1.1) in placebo, resulting in an estimated treatment difference (ETD) of -13.07 percentage points (95% CI, -15.61 to -10.52; P<0.001) using the treatment policy estimand. The trial product estimand yielded similar findings.

A significantly higher percentage of participants attained a minimum 5% decrease in body weight relative to baseline to week 68 with oral semaglutide (107 of 127, 84.3%) compared to placebo (11 of 64, 17.2%). This trend was consistent across both the in-trial and on-treatment periods, with 89.7% (105 of 117) in the semaglutide group and 17.5% (11 of 63) in the placebo subset reaching this threshold during the on-treatment period. The likelihood of achieving at least a 5% weight reduction was significantly higher in the oral semaglutide 50 mg group than in the inactive control group, across both treatment policy (odds ratio, 23.00; 95% CI, 10.28-51.42; P<0.001) and trial product estimands. The participants receiving oral semaglutide were markedly prone compared to those on placebo to achieve a decrease of 10%, 15%, or 20% or more in body weight from baseline to week 68, according to both the treatment policy and trial product estimand.

Beyond body weight loss, the participants taking semaglutide orally experienced greater improvements in IWQOL-Lite-CT physical function scores compared to those on placebo. Nevertheless, the difference failed to reach statistical significance (treatment policy estimand; ETD, 5.30; 95% CI, -0.01 to 10.62; P=0.05). Favorable changes were seen from onset to 68 weeks with oral semaglutide versus placebo across multiple supportive secondary endpoints, such as BMI, absolute body weight, abdominal girth, HbA1c, systolic and diastolic blood pressure, lipid level, and hsCRP levels, for both the treatment policy and trial product estimands. The participants who underwent baseline CT scans experienced more favorable changes in visceral adipose tissue area and subcutaneous adipose tissue area with oral semaglutide compared to placebo at week 68, according to the treatment policy estimand. Among those with type 2 diabetes at baseline, nearly three-quarters of oral semaglutide recipients with available data at week 68 had reverted to either prediabetes (15 of 33, 45.5%) or normoglycemia (10 of 33, 30.3%), compared to only one of 16 (6.3%) in the placebo group who reverted to prediabetes [12].

Safety:* *Adverse events occurred in 122 of 134 participants (91.0%) receiving oral semaglutide and 58 of 66 (87.8%) receiving placebo. The most common adverse events were gastrointestinal disorders, reported by 85 of 134 (63.4%) in the subset taking semaglutide orally and 23 of 66 (34.8%) in the inactive control group, as well as COVID-19 infection. Most of the digestive system symptoms were of mild to moderate severity and mostly happened during the dose-up titration phase. The safety of oral semaglutide in the OASIS 2 trial was comparable with that in the OASIS 1 trial [10]. Table 2 shows some of the adverse effects experienced.

**Table 2:** Adverse events in the OASIS 2 trial during the on-treatment period in the safety analysis Table adapted from OASIS 2 by Blessing Ojinna [12] n, number of participants; OASIS, Oral Semaglutide in Adults with Overweight or Obesity; NA, not available

Adverse event	Semaglutide 50 mg, tablet (n=134)	Semaglutide 50 mg, tablet (n=134)	Semaglutide 50 mg, tablet (n=134)	Placebo (n=66)	Placebo (n=66)	Placebo (n=66)
	Participants, number (%)	Events, number	Event rate	Participants, number (%)	Events, number	Event Rate
Any adverse event	122 (91.0%)	590	326.2	58 (87.9%)	239	258.8
Serious adverse events	8 (6.0%)	8	4.4	6 (9.1%)	7	7.6
Adverse events leading to trial product discontinuation	6 (4.5%)	7	3.9	0	NA	NA
Fatal events	0	NA	NA	0	NA	NA
Adverse events reported in >10% of the participants	Adverse events reported in >10% of the participants	Adverse events reported in >10% of the participants	Adverse events reported in >10% of the participants	Adverse events reported in >10% of the participants	Adverse events reported in >10% of the participants	Adverse events reported in >10% of the participants
COVID-19	41 (30.6%)	44	24.3	22 (33.3%)	22	23.8
Nausea	34 (25.4%)	46	25.4	5 (7.6%)	5	5.4
Constipation	28 (20.9%)	31	17.1	6 (9.1%)	7	7.6
Diarrhea	22 (16.4%)	32	17.7	6 (9.1%)	8	8.7
Vomiting	20 (14.9%)	34	18.8	2 (3.0%)	2	2.2
Pyrexia	16 (11.9%)	21	11.6	10 (15.2%)	15	16.2
Nasopharyngitis	15 (11.2%)	19	10.5	15 (22.7%)	17	18.4
Safety areas of interest	Safety areas of interest	Safety areas of interest	Safety areas of interest	Safety areas of interest	Safety areas of interest	Safety areas of interest
Gastrointestinal tract	85 (63.4%)	209	115.5	23(34.8%)	46	49.8
Dysesthesia	12 (9.0%)	17	9.4	1 (1.15%)	1	1.1
Cardiovascular disorders	10 (7.5%)	13	6.8	3 (4.5%)	3	3.2
Benign and malignant neoplasms	8 (6.0%)	11	5.8	3 (4.5%)	3	3.2
Allergic reactions	6 (4.5%)	10	5.5	7 (10.6%)	10	10.8
Psychiatry disorders	6 (4.5%)	6	3.3	2 (3.0%)	2	2.2
Hypoglycemia	1 (3.1%)	1	2.2	0	NA	NA

Summary of OASIS 3 Trial Evidence

OASIS 3 assessed the effectiveness and tolerability of semaglutide 50 mg taken orally daily versus placebo among 200 adults from China who were obese or overweight, monitored for 44 weeks, at hospitals in Beijing, Chongqing, Guangdong, Henan, Hubei, Hunan, Jiangsu, Shandong, Shanghai, and Tianjin. They included men and women from age 18 and above who had a BMI of at least 28 kg/m² or 24 kg/m² and above, with no fewer than one weight-associated complication, which could be type 2 diabetes, hyperlipidemia, sustained high blood pressure, sleep apnea, and cardiac disease, and who had a minimum of one failed nutritional management for weight loss. They excluded people who had a history of type 1 or type 2 diabetes at screening, who were treated with glucose-lowering agents within 90 days prior to screening and who had HbA1c greater than or equal to 6.5% at screening. In addition, they excluded patients with type 2 diabetes mellitus, who had uncontrolled and potentially unstable diabetic retinopathy or neuropathy and who had renal impairment with an estimated glomerular filtration rate (eGFR) of less than 30 mL/minute.

The study was a randomized, controlled phase 3 trial with a quadruple-blind design including study enrollee, care provider, researcher, and endpoint evaluator, with a simultaneous group allocation of treatment versus placebo. The participants received semaglutide tablets orally once daily with a dose-titration design as follows for a 44-week period: 3 mg administered from weeks 0 to 4, 7 mg administered from weeks 5 to 8, 14 mg administered from weeks 9 to 12, 25 mg administered from weeks 13 to 16, and 50 mg administered from weeks 17 to 44.

Their primary outcome measure was body weight variation relative to baseline, measured in percentage from zero to 44 weeks, and the number of participants who attained a 5% or above weight loss by the end of treatment week 44. Secondary endpoints comprised the amount of study subjects who attained a decrease in their body weight of 10%, 15%, and 20%; change in Impact of Weight on Quality of Life-Lite Clinical Trials Version (IWQOL-Lite-CT), functional ability domain, total score; and variation in BMI, abdominal circumference, blood pressure, glycated hemoglobin, fasting plasma glucose and fasting serum insulin, lipid profile, thyroid-stimulating hormone (TSH), high-sensitivity C-reactive protein, and treatment emergent adverse effects at peaks 44 and 51 [15]. The trial is registered in ClinicalTrials.gov, and results are pending publication in a peer-reviewed journal.

Summary of OASIS 4 Trial Evidence

OASIS 4 lasted for 71 weeks, as a multicenter, randomly assigned, placebo comparison, and double-masking study performed across four countries (Canada, Germany, Poland, and the United States) in 22 sites. A total of 307 adults without diabetes were enrolled and randomized, from October 2022 to May 2024, with 205 participants assigned to oral semaglutide and 102 to placebo. Eligible subjects were adults aged 18 or older without diabetes who had a BMI of ≥27 kg/m² or higher, with a minimum of one obesity-related complication (hypertension, dyslipidemia, sleep apnea, or cardiovascular disease), and who had attempted but not succeeded in losing weight through diet. Baseline demographic and clinical characteristics were reported to be balanced between treatment groups. The study population was predominantly female participants (78.8%) and White (91.5%). The average body weight among participants was 105.9 kg, with a mean BMI of 37.6. The average abdominal circumference measured 113.9 cm, and the average HbA1c level was 5.7%.

The OASIS 4 clinical trial has demonstrated substantial efficacy of 25 mg oral semaglutide, as an alternative to subcutaneous semaglutide (2.4 mg) and 50 mg oral semaglutide for individuals with overweight or obesity [16].

In the study, the participants were randomized in a 2:1 ratio to either a semaglutide tablet or a placebo, alongside a healthy behavior strategy. Randomization was conducted centrally using an interactive online response system, without stratification. Lifestyle interventions included dietary counseling targeting daily caloric reduction of 500 kcal and recommendations for a minimum of 150 minutes of exercise weekly. Oral semaglutide was administered daily, starting with 3 mg and up-titration every four weeks (week 4, 7 mg; week 8, 14 mg) to a target maintenance dose of 25 mg by week 12. The maintenance phase continued until week 64, followed by a seven-week follow-up. Dose reductions were permitted for tolerability, with attempts to re-escalate encouraged. Study medication was taken in the morning after overnight fasting, with at least 120 mL of water, within 30 minutes before food or other oral medications. The use of non-study obesity medications during the trial was prohibited.

Results:* *Two monitoring phases were established: the study phase, which spanned from the date of random assignment to the participant's final contact with the study location, and the therapeutic period, which extended from the date of the initial dosing of semaglutide or placebo until the date of last administration, plus three days for efficacy analyses or 49 days for safety analyses, excluding any temporary interruptions. A total of 290 participants (94.5%) completed the trial by attending the week 71 visit, including 95.6% in the subset receiving semaglutide orally and 92.2% in the placebo subset. Among those who finished the treatment regimen with oral semaglutide, 136 participants (81.4%) received a final dose of 25 mg, 14 (8.4%) received 14 mg, and 16 (9.6%) received a dose of less than 14 mg.

In this randomized trial of 307 participants with obesity, retention and completion rates were high (>79% on treatment at 64 weeks). Baseline characteristics were well balanced (mean BMI: 37.6 kg/m²), and oral semaglutide produced a significantly greater mean weight reduction at 64 weeks than placebo (-13.6% versus -2.2%; estimated treatment difference, -11.4 percentage points; 95% CI -13.9 to -9.0; P<0.001). Principal study outcomes were the percentage variation in body weight and attaining ≥5% weight reduction at 64 weeks. Confirmatory secondary outcomes included proportions achieving ≥10%, ≥15%, and ≥20% weight loss and change in physical function (IWQOL-Lite-CT). In addition to weight reduction, oral semaglutide was associated with significant improvements in physical function. The participants treated with oral semaglutide had greater increases in the IWQOL-Lite-CT physical function score than those receiving placebo, and a higher proportion achieved clinically meaningful improvements.

Treatment was also associated with favorable changes in cardiometabolic risk factors, including measures of glycemic control, lipid metabolism, and systemic inflammation among participants with prediabetes; most reverted to normoglycemia. Effects on blood pressure were modest and did not differ meaningfully from placebo.

Safety:* *Oral semaglutide was linked to an increased overall rate of undesirable events relative to placebo, largely driven by digestive diseases. Many gastrointestinal treatment-related events with semaglutide were nonserious, minimal to intermediate in intensity, temporary, and observed most frequently during or shortly after dose escalation. Permanent discontinuation due to gastrointestinal adverse events was uncommon.

Gastrointestinal events, particularly nausea and vomiting, accounted for the highest reporting frequency and occurred commonly with oral semaglutide. The rate of serious adverse events was low and did not differ between oral semaglutide and placebo. No deaths were reported. Overall, oral semaglutide demonstrated an acceptable tolerability profile similar to the GLP-1 receptor agonist group. Table 3 shows a summary of safety outcomes.

**Table 3:** Adverse events in the OASIS 4 trial Table adapted from OASIS 4 by Rabia Channa and Blessing Ojinna [16] n, number of participants; OASIS, Oral Semaglutide in Adults with Overweight or Obesity; GI, gastrointestinal

Adverse event	Semaglutide 25 mg, tablet (n=134)	Semaglutide 25 mg, tablet (n=134)	Semaglutide 25 mg, tablet (n=134)	Placebo (n=66)	Placebo (n=66)	Placebo (n=66)
	Participants, number (%)	Events, number	Event rate	Participants, number (%)	Events, number	Event rate
Any adverse event	190 (93.1%)	1239	493.5	87 (85.3%)	432	355.9
Serious adverse events	8 (3.9%)	17	6.8	9 (8.8%)	13	10.7
Adverse events leading to trial product discontinuation	14 (6.9%)	14	5.6	6 (5.9%)	6	4.9
Fatal events	0	0	0	0	0	0
GI disorder	7 (3.4%)	7	2.8	2 (2.0%)	2	16
Adverse events reported in >10% of the participants	Adverse events reported in >10% of the participants	Adverse events reported in >10% of the participants	Adverse events reported in >10% of the participants	Adverse events reported in >10% of the participants	Adverse events reported in >10% of the participants	Adverse events reported in >10% of the participants
Diarrhea	36 (17.6)	61	24.3	9 (8.8%)	10	8.2
Nausea	95 (46.6)	157	62.5	19 (18.6%)	27	22.2
Constipation	41 (20.1%)	59	23.5	10 (9.8%)	11	9.1
Vomiting	22 (16.4%)	32	17.7	6 (9.1%)	8	8.7
Dyspepsia	37 (18.1%)	50	19.9	9 (8.8%)	11	9.1
COVID-19	42 (20.6%)	46	18.3	18 (17.6%)	19	15.7
Nasopharyngitis	43 (21.1%)	59	23.5	27 (26.5%)	40	33.0
Headache	24 (11.8%)	35	13.9	9 (8.8%)	10	8.2
Eructation	21 (10.3%)	23	9.2	2 (2.0%)	2	1.6

The demographic and result summary of the four OASIS Trials is shown in Table 4.

**Table 4:** Comparative summary of OASIS trials Table created by Blessing Ojinna using data from OASIS 1, 2, 3, and 4 [10,12,15,16] CI, confidence intervals; OASIS, Oral Semaglutide in Adults with Overweight or Obesity

Trial	Number of participants	Diabetes present or not	Demographic area	Population	Dose	Duration	Primary endpoint	Key results
OASIS 1	667	No diabetes	Asia, Europe, and North America	Obesity/overweight (no diabetes), multicountry	Semaglutide 50 mg	68 weeks	Percentage weight loss	-15.1% versus -2.4% (semaglutide versus placebo) (95% CI, -14.2 to -11.3; p<0.0001)
OASIS 2	201	Diabetes present	Japan and South Korea	East Asian (±diabetes) and Japan/Korea	Semaglutide 50 mg	68 weeks	Percentage weight loss	~-14.3% versus -1.3% placebo (semaglutide versus placebo) (95% CI, -15.61 to -10.52; P<0.001)
OASIS 3	200	Diabetes present	Chinese adults	Overweight/obesity regional cohort (e.g., China, ±diabetes), 16 sites	Semaglutide 50 mg	44 weeks	Percentage weight loss	Details pending full publication
OASIS 4	307	No diabetes	Canada, Germany, Poland, and the United States	Obesity/overweight (no diabetes), four countries	Semaglutide 25 mg	64 weeks	Percentage weight loss	~13.6% mean loss versus ~2.2% (semaglutide versus placebo) (95% CI, -13.9 to -9.0; p<0.001)

Comparing and compiling the evidence for semaglutide

The clinical trials mentioned above have clearly demonstrated that the efficacy of oral semaglutide in reducing body weight is comparable to that of semaglutide injected subcutaneously. In the OASIS 1, 2, and 4 trials, once-daily oral semaglutide at doses of 50 mg or 25 mg demonstrated superiority relative to the inactive control in reducing weight in patients who were overweight or obese, with or without T2DM [10,12,16]. In this section, we compared the results from OASIS 1, 2, and 4 trials based on the trial findings as demonstrated in Table 4, and individual results from these trials are shown in Table 5.

**Table 5:** Summary of OASIS trial 1, 2, and 4 results Table created by Blessing Ojinna and Sara Tariq using data from OASIS 1, 2, and 4 [10,12,16]

Trial (n)	Dose and comparators	Estimated mean weight change (%)	Patients with weight loss of >5%	Patients with weight loss of >10%	Patients with weight loss of >15%	Patients with weight loss of >20%
OASIS 1 (n=667)	Oral semaglutide 50 mg	-15.1	85	69	54	34
	Placebo	-2.4	26	12	6	3
OASIS 2 (n=201)	Oral semaglutide 50 mg	-14.3	84.3	65.4	47.2	32.3
	Placebo	-1.3	17.2	9.4	0	0
OASIS 4 (n=307)	Oral semaglutide 50 mg	-13.6	79.2	63	50	29.7
	Placebo	-2.2	31.1	14.4	5.6	3.3

In the OASIS 1 trial, 50 mg oral semaglutide was associated with a -15.5% change in body weight from baseline, whereas patients in the placebo group reported a -2.5% change [8]. Similarly, when compared with semaglutide injections, a 2.4 mg dose was in close resemblance with 50 mg oral Wegovy in terms of safety profile and efficacy [9]. The same trend was observed again in OASIS 2 and 4 trials [12,16]. Another finding observed in the abovementioned trials was the increased weight loss with escalating oral semaglutide doses, depending upon the tolerability of the participant [10,12,16].

Mostly, the side effects reported in the oral semaglutide group involved the gastrointestinal system, including but not limited to nausea, vomiting, and diarrhea. Treatment discontinuation in the OASIS 1 trial due to GI tract adverse events was 4% in the group taking semaglutide orally and 2% in the placebo subset [10]. The incidence of hypoglycemia was very low in all OASIS trials, with results reported [10,12].

The route of administration seems to be the only apparent difference between oral and injectable semaglutide, as their efficacy is almost similar. A study from 2012 shows that patients prefer taking oral tablets compared to subcutaneous injections due to less pain and the ease of administration [17]. Based on these results, patients would preferably go for a tablet rather than an injection. Therefore, the Food and Drug Administration (FDA) has approved the use of oral Wegovy as a weight loss drug for sustained weight control among adults with obesity status or above-normal weight and a minimum of one or more weight-associated comorbidities. The future inclusion of results from the OASIS 3 trial will allow for a more comprehensive and conclusive evaluation.

Discussion

Individuals with obesity face a reduced life expectancy and a heightened predisposition to developing comorbid conditions, including type 2 diabetes and heart and blood vessel disorders. Pharmacological therapies serve as valuable adjuncts to lifestyle interventions, as many people find it challenging to achieve and sustain weight loss through diet and exercise alone [18,19].

Despite the proven efficacy and safety of injectable GLP-1 medications, their utilization remains very low. Fewer than 2% of eligible patients receive prescriptions, even though many would benefit from treatment [20,21]. The access to an oral GLP-1 receptor agonist with proven weight loss effects offers individuals with obesity and their doctors a valuable new option for managing obesity and related comorbidities. Patients can now choose between subcutaneous or oral administration based on individual choices, while doctors may prefer an oral therapeutic agent, and this may be easier to prescribe in office settings [22,23].

Individuals may favor oral formulations over the injectables due to the fear of injections and the risk of injection-site response. Furthermore, oral agents typically do not require a refrigerated supply chain, which may expand access to obesity care in areas where refrigeration is limited [24,25].

**Figure 1:**
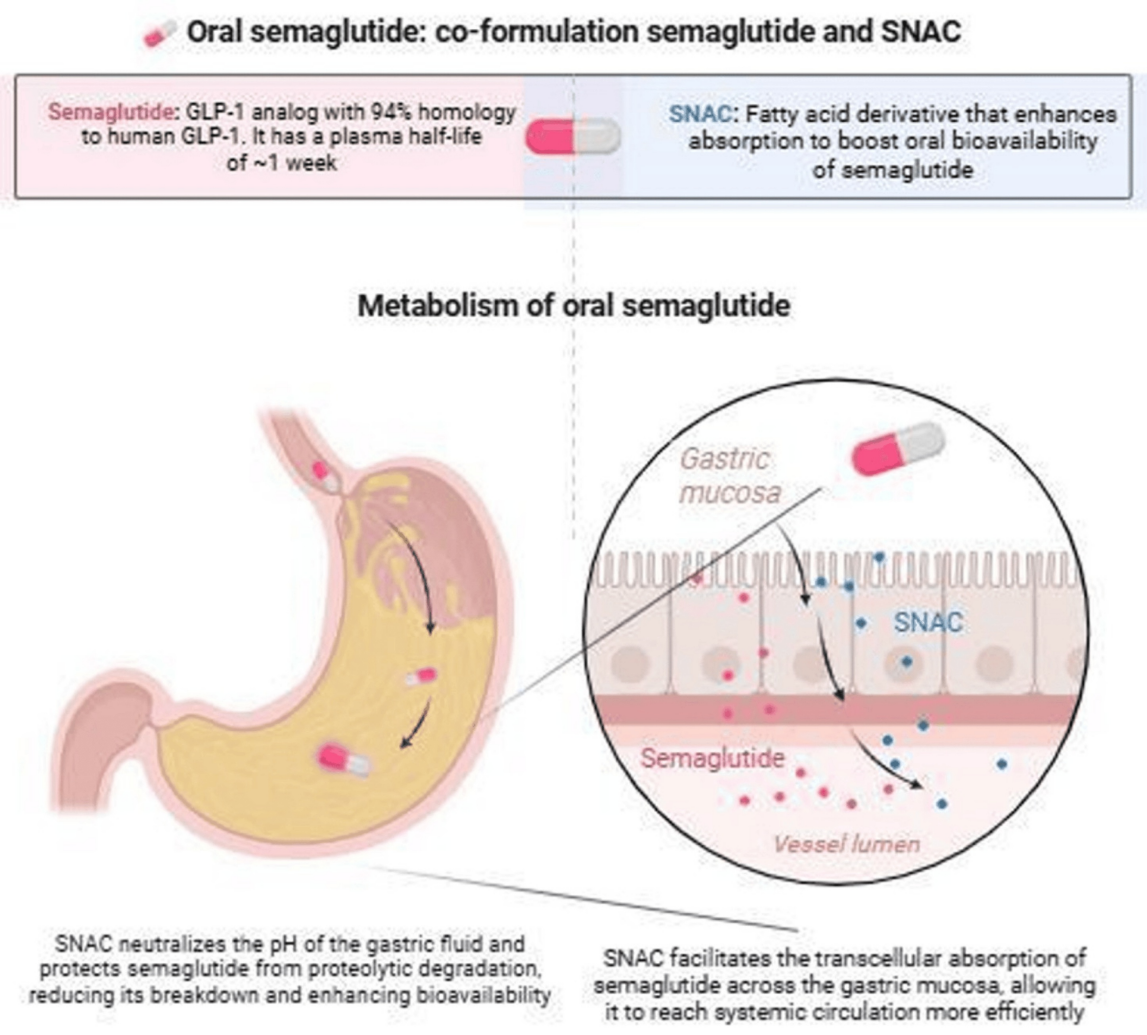
Bioavailability schematic of the oral tablet absorption process in the stomach Created by Sara Tariq on BioRender (BioRender, Toronto, Canada) SNAC, sodium N-(8-[2-hydroxylbenzoyl]amino) caprylate; GLP-1, glucagon-like peptide-1

Oral GLP-1 agonists have been made possible through technological advances that allow the successful delivery of a pharmacological peptide through an oral route, and the oral formulation of semaglutide includes the bioenhancer sodium N-(8-[2-hydroxylbenzoyl]amino) caprylate (SNAC) to improve its bioavailability. Oral semaglutide is absorbed primarily in the stomach, but its peptide structure makes it vulnerable to degradation in the acidic gastric environment and by digestive enzymes. Coformulation with the absorption enhancer SNAC (an N-acetylated salicylic acid-derived amino acid used within the Eligen carrier system) overcomes these barriers and enables effective gastric absorption, making oral semaglutide the first FDA-approved peptide drug formulated with SNAC. Imaging studies, including gamma scintigraphy, have demonstrated site-directed release and uptake in the stomach. SNAC facilitates absorption through multiple mechanisms: It transiently raises the local pH around the tablet, thereby protecting semaglutide from acid-mediated degradation and enzymatic breakdown; it reduces peptide self-association (oligomerization), improving availability for absorption; and it enhances transcellular transport by increasing epithelial membrane fluidity and permeability. Importantly, SNAC has minimal effects on tight junctions, indicating that absorption occurs predominantly via a transcellular rather than paracellular pathway. Effective uptake depends on close spatial association between semaglutide and SNAC, achieved through coformulation that ensures simultaneous release at the gastric epithelial surface and sustained local drug concentration [26]. Figure 1 is an illustrative diagram of the oral tablet absorption process in the stomach, showing how the SNAC enhancer facilitates semaglutide transport across the gastric epithelium.

The dose of oral semaglutide (50 mg daily) is significantly higher than the subcutaneous dose (2.4 mg weekly) due to a difference in bioavailability. Oral semaglutide is absorbed poorly compared to subcutaneous (SC) injections, so the oral dose has to be much higher to achieve similar systemic exposure. Even with the SNAC absorption enhancers, <1% of the oral dose is absorbed. Some of the reasons why the oral formulations have a poor bioavailability include the degradation of the medication by gastric enzymes and the stomach's acidic pH. The subcutaneous dose bypasses the gastrointestinal tract and delivers the drug directly into the systemic circulation with a bioavailability of ~80%-90% [27].

**Figure 2:**
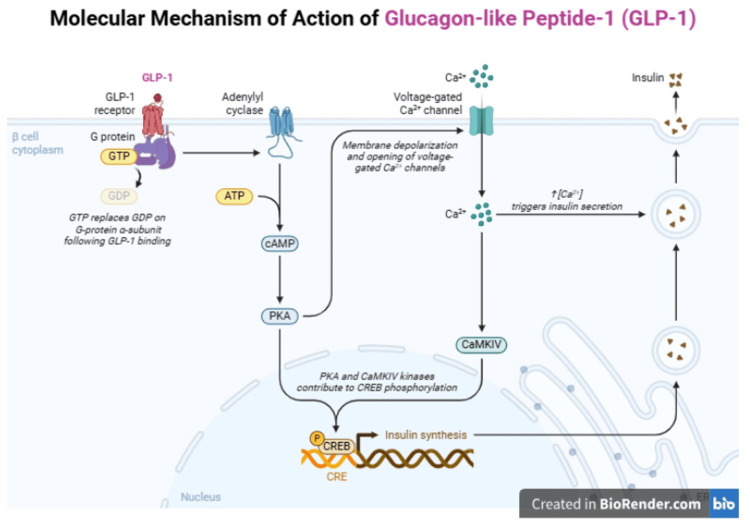
Illustrative schematic of GLP-1 receptor agonist mechanism of action Created by Rabia Channa on BioRender GTP, guanosine triphosphate; GDP, guanosine diphosphate; cAMP, cyclic adenosine monophosphate; ATP, adenosine triphosphate; PKA, protein kinase A; CRE, cAMP response elements

In a phase 2 dose-finding study, the participants with type 2 diabetes who took oral semaglutide 40 mg once daily experienced an average weight loss of 5.7 kg over 26 weeks, regardless of their baseline weight status. The safety profile is similar to other GLP-1 analogs [28]. Figure 2 shows the mechanism of action of GLP-1 receptor agonists.

A 50 mg dosing of oral semaglutide is based on modeling data suggesting that this higher dose would enhance weight loss while maintaining a safety outcome. Consequently, 50 mg was selected for obesity treatment studies. This dose is higher than the 2.4 mg once-weekly dose used for the subcutaneous formulation due to differences in bioavailability between the two delivery methods [10]. Exposure-response analyses comparing 14 mg daily semaglutide taken orally with subcutaneous semaglutide 1 mg weekly in patients with type 2 diabetes have shown that the association across semaglutide blood concentration and its effects on long-term glycemic markers and body mass is nearly identical, irrespective of how the medication is administered [29].

Among individuals with type 2 diabetes who had suboptimal control with lifestyle modifications alone, oral semaglutide (14 mg) monotherapy led to a demonstrable reduction in body weight compared to inactive control (at week 26; ETD, -2.3 kg; p<0.001). In contrast, the 3 mg and 7 mg doses of oral semaglutide did not differ significantly from placebo (PIONEER trial) [30]. Oral Wegovy substantially reduces the probability of notable undesirable cardiac events compared to placebo for patients with diabetes and documented coronary artery disease, chronic renal disease, or both, without increasing serious adverse events [31].

Statistical Analysis

The OASIS trial program provides compelling and consistent evidence for the superiority of semaglutide taken orally over placebo, as seen in their individual statistical analysis. Findings from the statistical evaluation of the coprimary and key confirmatory secondary outcome measures were presented with two-sided 95% confidence intervals and corresponding p values (with superiority defined as p<0.05). For the coprimary outcome, superiority was required to be established for each outcome independently at a significance threshold of 0.05 before oral semaglutide 50 mg could be considered superior to placebo. P values were not provided for the supplementary secondary outcomes because these analyses did not account for multiplicity. Accordingly, findings from these endpoints should be interpreted cautiously and not considered conclusive evidence of therapeutic outcomes.

In OASIS 1, oral semaglutide 50 mg produced a mean body weight reduction of -15.1% (SE: 0.5) versus -2.4% (0.5) with placebo, yielding an estimated treatment difference (ETD) of -12.7 percentage points (95% CI, -14.2 to -11.3; p<0.0001). In OASIS 2, oral semaglutide 50 mg produced a mean reduction of -14.3% (SEM: 0.8) compared to -1.3% (1.1) seen in placebo (ETD, -13.07 percentage points; 95% CI, -15.61 to -10.52; P<0.001). In OASIS 4, the calculated average percentage variation in body weight from baseline to 64 weeks was -13.6% in the semaglutide cohort compared to -2.2% in the placebo cohort (ETD, -11.4 percentage points; 95% confidence interval, -13.9 to -9.0; p<0.001) [10,12,16].

This alignment of statistical and clinical significance across three independent trials, two dose levels, and multiple ethnic populations provides a robust foundation for the superiority claim and supports the therapeutic value of oral semaglutide in the management of overweight and obesity.

The newly approved oral semaglutide formulations of 25 mg and 50 mg (Wegovy) for weight management differ from Rybelsus primarily by their higher dosing. Data from the OASIS trials demonstrated that these higher doses achieved significantly greater body weight reduction than oral semaglutide 14 mg (Rybelsus) in previous studies, supporting their use in patients requiring more substantial weight loss.

**Figure 3:**
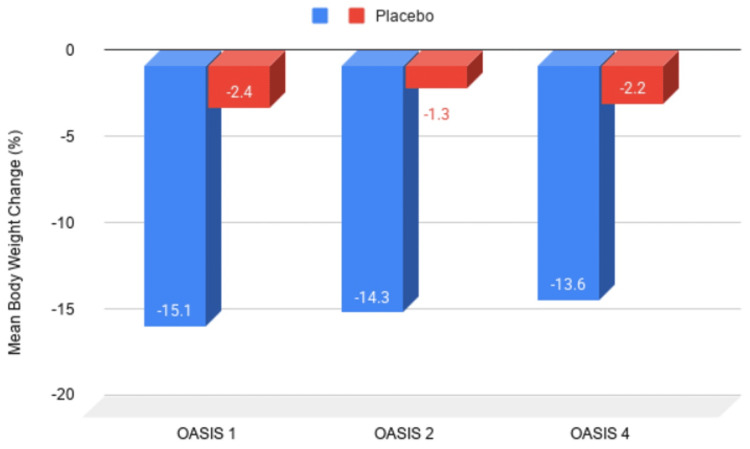
Illustrative bar chart comparing the percentage of body weight change in the OASIS trials Created by Sara Tariq in Google Sheets (Google, Inc., Mountain View, CA) OASIS: Oral Semaglutide in Adults with Overweight or Obesity

The OASIS trials have demonstrated that higher-dose oral semaglutide is effective in achieving significant body weight reduction, making it a viable alternative to injectable semaglutide for weight management. Figure 3 is an illustrative bar chart comparing the percentage of body weight change across OASIS 1 (-15.1%), OASIS 2 (-14.3%), and OASIS 4 (-13.6%) versus their respective placebos.

Adverse Events

As detailed above, in the OASIS 1, 2, and 4 trials, the participants taking oral semaglutide experienced more side effects than those taking a placebo. Mostly reported undesirable events were of the digestive system, such as queasiness, vomiting, watery stools, and difficult defecation. Mostly, these side effects manifested during the dose-titration phase and appeared transient and minimal to moderate and subsided without the discontinuation of the drug [10,12,16]. In the OASIS 4 trial, altered skin sensation (dysesthesia) was reported in 10 (4.9%) participants in the oral semaglutide group [16]. There were no reported pancreatitis events. Gallbladder-related disorders such as cholelithiasis were reported in 13 (4%) participants [10].

There were also concerns about developing COVID-19 infection in some participants (OASIS 1, 2, and 4) [10,12,16]. In addition, there were reports of nasopharyngitis in >10% of patients [12,16]. Other less commonly reported adverse effects were cardiovascular disorders and psychiatric and benign or malignant tumors [10]. Black box warnings include contraindications based on findings from animal studies, including personal and family history of multiple endocrine neoplasia disorder 2 (MEN2) and medullary thyroid carcinoma [32].

Limitations

There are a few limitations in this literature review. OASIS 3 trials have not been included in the comparison as their results have not been published yet officially in a peer-reviewed journal, which affects the conclusive outcome for all trials.

Therefore, the deducted conclusions cannot be considered definitive, and future studies are recommended to study the efficacy of semaglutide in depth, which includes the OASIS 3 trial. The participants in the OASIS 4 trial were mainly women (78.8%).

## Conclusions

Oral Wegovy has been approved for chronic weight loss management. In addition, it also reduces major adverse cardiovascular events in patients with documented cardiovascular disease who are overweight and obese. Oral Wegovy (semaglutide) has a comparable efficacy and safety profile to semaglutide injectables for the long-term management of weight reduction and can be an effective alternative due to better tolerability, compliance, and lower cost. It may positively impact patients' quality of life by achieving outcomes similar to those with oral pills, without the pain of injections. Though we have the oral options, we are still bound by the insurance barriers, which can affect weight management, further growing the obesity burden. Therefore, it is recommended to devise a cost-effective oral Wegovy prescription treatment plan tailored with insurance to help clinicians decide the preferred option for their patients.

## References

[1] (2026). Obesity and overweight. https://www.who.int/news-room/fact-sheets/detail/obesity-and-overweight.

[2] (2026). Adult obesity facts. https://www.cdc.gov/obesity/adult-obesity-facts/index.html.

[3] Kinlen D, Cody D, O'Shea D (2018). Complications of obesity. QJM.

[4] Okunogbe A, Nugent R, Spencer G, Powis J, Ralston J, Wilding J (2022). Economic impacts of overweight and obesity: current and future estimates for 161 countries. BMJ Glob Health.

[5] Cawley J, Biener A, Meyerhoefer C, Ding Y, Zvenyach T, Smolarz BG, Ramasamy A (2021). Direct medical costs of obesity in the United States and the most populous states. J Manag Care Spec Pharm.

[6] Isaacs D, Prasad-Reddy L, Srivastava SB (2016). Role of glucagon-like peptide 1 receptor agonists in management of obesity. Am J Health Syst Pharm.

[7] Krajnc M, Kuhar N, Koceva A (2025). Oral semaglutide for the treatment of obesity: a retrospective real-world study. Front Endocrinol (Lausanne).

[8] Elmaleh-Sachs A, Schwartz JL, Bramante CT, Nicklas JM, Gudzune KA, Jay M (2023). Obesity management in adults: a review. JAMA.

[9] Bergmann NC, Davies MJ, Lingvay I, Knop FK (2023). Semaglutide for the treatment of overweight and obesity: a review. Diabetes Obes Metab.

[10] Knop FK, Aroda VR, do Vale RD (2023). Oral semaglutide 50 mg taken once per day in adults with overweight or obesity (OASIS 1): a randomised, double-blind, placebo-controlled, phase 3 trial. Lancet.

[11] Wilding JP, Batterham RL, Calanna S (2021). Once-weekly semaglutide in adults with overweight or obesity. N Engl J Med.

[12] Kadowaki T, Heftdal LD, Ko HJ, Overvad M, Shimomura I, Thamattoor UK, Kim KK (2025). Oral semaglutide in an East Asian population with overweight or obesity, with or without type 2 diabetes: the OASIS 2 randomized clinical trial. JAMA Intern Med.

[13] Kim KK, Haam JH, Kim BT (2023). Evaluation and treatment of obesity and its comorbidities: 2022 update of clinical practice guidelines for obesity by the Korean Society for the Study of Obesity. J Obes Metab Syndr.

[14] Ogawa W, Hirota Y, Miyazaki S (2024). Definition, criteria, and core concepts of guidelines for the management of obesity disease in Japan. Endocr J.

[15] (2026). Research study looking at how well semaglutide tablets taken once daily work in Chinese adults who are above a healthy weight range (OASIS 3) (OASIS 3). https://clinicaltrials.gov/study/NCT05890976.

[16] Wharton S, Lingvay I, Bogdanski P (2025). Oral semaglutide at a dose of 25 mg in adults with overweight or obesity. N Engl J Med.

[17] Quante M, Thate-Waschke I, Schofer M (2012). What are the reasons for patient preference? A comparison between oral and subcutaneous administration (Article in German). Z Orthop Unfall.

[18] Garvey WT, Mechanick JI, Brett EM (2016). American Association of Clinical Endocrinologists and American College of Endocrinology comprehensive clinical practice guidelines for medical care of patients with obesity. Endocr Pract.

[19] (2016). Erratum. Obes Facts.

[20] Saxon DR, Iwamoto SJ, Mettenbrink CJ (2019). Antiobesity medication use in 2.2 million adults across eight large health care organizations: 2009-2015. Obesity (Silver Spring).

[21] Claridy MD, Czepiel KS, Bajaj SS, Stanford FC (2021). Treatment of obesity: pharmacotherapy trends of office-based visits in the United States from 2011 to 2016. Mayo Clin Proc.

[22] Enright C, Thomas E, Saxon DR (2023). An updated approach to antiobesity pharmacotherapy: moving beyond the 5% weight loss goal. J Endocr Soc.

[23] Elangovan A, Shah R, Smith ZL (2021). Pharmacotherapy for obesity-trends using a population level national database. Obes Surg.

[24] Gallwitz B, Giorgino F (2021). Clinical perspectives on the use of subcutaneous and oral formulations of semaglutide. Front Endocrinol (Lausanne).

[25] Boye K, Ross M, Mody R, Konig M, Gelhorn H (2021). Patients' preferences for once-daily oral versus once-weekly injectable diabetes medications: the REVISE study. Diabetes Obes Metab.

[26] Solis-Herrera C, Kane MP, Triplitt C (2024). Current understanding of sodium N-(8-[2-hydroxylbenzoyl] amino) caprylate (SNAC) as an absorption enhancer: the oral semaglutide experience. Clin Diabetes.

[27] Aroda VR, Blonde L, Pratley RE (2022). A new era for oral peptides: SNAC and the development of oral semaglutide for the treatment of type 2 diabetes. Rev Endocr Metab Disord.

[28] Davies M, Pieber TR, Hartoft-Nielsen ML, Hansen OK, Jabbour S, Rosenstock J (2017). Effect of oral semaglutide compared with placebo and subcutaneous semaglutide on glycemic control in patients with type 2 diabetes: a randomized clinical trial. JAMA.

[29] Suran M (2023). As Ozempic’s popularity soars, here’s what to know about semaglutide and weight loss. JAMA.

[30] Aroda VR, Rosenstock J, Terauchi Y (2019). PIONEER 1: randomized clinical trial of the efficacy and safety of oral semaglutide monotherapy in comparison with placebo in patients with type 2 diabetes. Diabetes Care.

[31] McGuire DK, Marx N, Mulvagh SL (2025). Oral semaglutide and cardiovascular outcomes in high-risk type 2 diabetes. N Engl J Med.

[32] Singh G, Krauthamer M, Bjalme-Evans M (2022). Wegovy (semaglutide): a new weight loss drug for chronic weight management. J Investig Med.

